# Surgical outcomes of total duct excision in the diagnosis and management of nipple discharge

**DOI:** 10.1308/rcsann.2022.0093

**Published:** 2024-03-18

**Authors:** K Ward, G Selvarajah, H Al-Omishy, M Sait, HN Khan, K McEvoy, S Robertson

**Affiliations:** ^1^ University of Birmingham, UK; ^2^ University Hospitals Coventry and Warwickshire NHS Trust, UK

**Keywords:** Total duct excision, Breast cancer, Nipple discharge, Invasive ductal carcinoma

## Abstract

**Introduction:**

Total duct excision (TDE) is performed for the diagnosis and management of nipple discharge. The Association of Breast Surgery’s recent guidelines recommend considering diagnostic surgery for single-duct, blood-stained or clear nipple discharge, and for symptomatic management.

**Methods:**

We retrospectively reviewed the diagnostic and surgical outcomes of all cases of TDE between January 2013 and November 2019.

**Results:**

In total, 259 TDEs were carried out: 219 for nipple discharge, 29 for recurrent mastitis, 3 for screening abnormalities and 8 for breast lumps. Of the nipple discharge group, 121 had blood-stained discharge. Mean patient age was 52 years (range 19–81). Median follow-up time was 45 months (interquartile range 24–63). The following cases were identified on histopathology: 236 benign breast changes, 10 atypical ductal hyperplasia, 4 lobular carcinoma in situ, 2 low-grade ductal carcinoma in situ (DCIS), 3 intermediate-grade DCIS, 2 high-grade DCIS and 2 invasive ductal carcinomas. In total, 3.5% of patients who underwent TDE had a diagnosis of DCIS or invasive carcinoma. Blood-stained discharge was associated with a significant increase in risk of DCIS or carcinoma compared with other nipple discharge colours (*p* = 0.043). The most common complications of TDE were infection, poor wound healing and haematoma. Nipple discharge recurred in 14.2% of cases.

**Conclusions:**

TDE can be considered for the diagnostics and management of nipple discharge. Blood-stained nipple discharge increases the risk of DCIS or malignancy, but the majority of the time TDE reveals benign breast pathology.

## Introduction

Nipple discharge is a frequent cause for presentation to breast clinic,^1^ and affects up to 80% of women of reproductive age.^2^ There are both physiological and pathological causes of nipple discharge including: pregnancy, hyperprolactinaemia, duct ectasia and recurrent mastitis.^1^ Because nipple discharge has traditionally been considered a possible sign of breast cancer, further assessment is routinely required for persistent and unexpected symptoms.^3^

Total duct excision (TDE) has traditionally been used in the diagnostics and management of suspicious and troublesome nipple discharge. TDE allows for histological assessment of ductal tissue and to look for small areas of occult disease that are not seen on imaging.^4-8^ TDE also has a therapeutic advantage, stopping or reducing the nipple discharge.^8,9^ An alternative option is microdochectomy for single-duct discharge, but this study focuses on TDE, which has traditionally been our multidisciplinary team’s (MDT) recommendation (if the patient is not considering breastfeeding in the future). Particular suspicion arises with persistent, single-duct nipple discharge as up to 23% of these cases may be associated with breast malignancy.^4-8^ Discharge colour may have predictive value. In a study of 925 cases of surgical duct exploration for persistent nipple discharge, 30.3% of those with blood-stained discharge and 17.6% of those with clear discharge had a diagnosis of carcinoma.^8^ Cytology, galactography and mammary ductoscopy have been suggested as alternatives for the assessment of suspicious nipple discharge. These tests are often unavailable, are poorly validated and are neither sensitive nor specific.^10,11^

In January 2019, the Association of Breast Surgery (ABS) introduced new guidelines on the management of nipple discharge.^1^ These guidelines emphasise that single-duct nipple discharge is concerning, and requires thorough clinical assessment. If clinical assessment and imaging are normal, the incidence of malignancy is estimated at below 3%. If single-duct nipple discharge is blood-stained or clear in colour, TDE or microdochectomy should be considered for further diagnostics. Within this study, we assess the diagnostic and surgical outcomes of TDE at a single, UK-based breast surgery department.

## Methods

**Figure 1:**
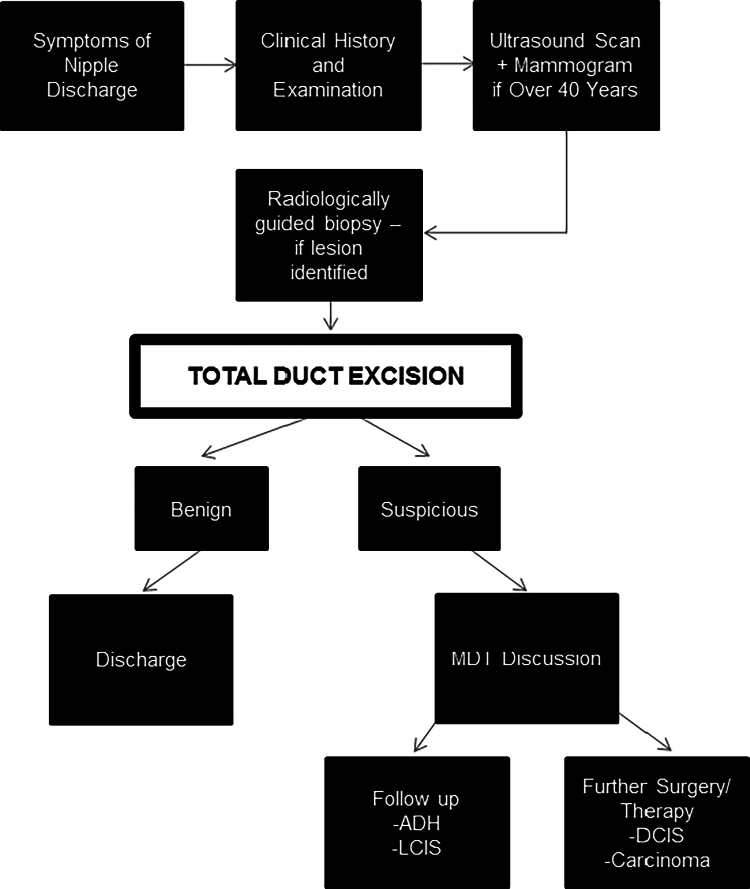
Local treatment algorithm for management of patients with suspicious nipple discharge. The flow diagram shows the local treatment pathway that patients with suspicious nipple discharge followed.

This is a retrospective study, conducted in the breast department of a busy university teaching hospital. All patients who underwent TDE or microdochectomy between January 2013 and November 2019 were included. The study was conducted in accordance with local research and ethics policies, and was approved locally by the research and development team (Project Reference/GF0289). All patients underwent a standardised work-up in the breast clinic consisting of history-taking, examination and breast imaging with ultrasound scan, plus mammography if aged 40 years or over (Figure 1). If a breast lesion was identified and accessible, radiologically guided biopsy was performed. Patients underwent TDE using a standard Hadfield’s technique. During this method, the major duct system is excised, leaving the nipple intact.^12^ All surgical specimens were sent to histopathology for diagnostics.

The following results were classified as ‘benign’: duct ectasia, papilloma (without atypia), fibroadenoma, fibrocystic changes, benign microcalcification, inflammation, scarring, normal breast tissue, benign breast tumours and usual type epithelial hyperplasia. The following diagnoses were classified into an ‘atypical, preinvasive or malignant’ category: atypical ductal hyperplasia (ADH), papilloma with atypia, lobular carcinoma in situ (LCIS), low-grade, intermediate-grade or high-grade ductal carcinoma in situ (DCIS) and established carcinoma.

All histology was discussed in a local MDT meeting. Patients with benign disease were reviewed post-operatively and discharged from the breast service. Patients with ADH or LCIS were followed-up with annual mammograms for 5 years. Patients with DCIS or invasive carcinoma underwent further treatment according to MDT recommendation, such as further surgery or adjuvant treatment, and surveillance mammography.

### Statistical analysis

Statistical analysis was carried out using Microsoft Excel and SPSS Statistics. The rate of malignant disease was compared between imaging findings and nipple discharge colour groups, using a two-tailed Fisher’s exact test. A logistic regression model was used to assess the association between age and breast malignancy on TDE. The level of significance for all statistical tests was taken as *p* < 0.05.

## Results

### Population

In total, 259 cases of TDE were performed, 257 in female patients, and 2 in male patients. Mean patient age was 52 years (range 19–81). Median follow-up time was 45 months (interquartile range 24–63).

### Surgical indication

In total, 219 patients (84.6%) underwent TDE for nipple discharge. Of these, 208 were TDE operations alone and 11 were done simultaneously with local excision biopsy, owing to abnormal findings on imaging or clinical examination. Twenty-nine patients (11.2%) underwent TDE for recurrent mastitis. Eleven patients (4.2%) underwent TDE for further diagnosis of a suspicious breast lump or thickening identified in breast clinic or on routine breast screening; two of which were performed with an additional excision biopsy. Twenty-six patients in this series had undergone previous ipsilateral TDE surgery, and underwent a repeat procedure.

### Nipple discharge colour

Nipple discharge colour was recorded for 207 cases. Nipple discharge was blood-stained in 124 cases (59.9%), clear in 41 cases (19.8%) and other colours (black, brown, cream, green, yellow or white) in 42 cases (20.3%). All cases of intermediate-grade DCIS, high-grade DCIS or invasive carcinomas were associated with blood-stained nipple discharge (Table 1).

**Table 1:** Demographics and outcomes of patients with intermediate to high-grade DCIS or carcinoma

No.	Discharge colour	Imaging findings	Diagnosis	Margins clear?
1	Blood	Dilated ducts	Intermediate-grade DCIS	No
2	Blood	Discrete lesion	Intermediate-grade DCIS	No
3	Blood	Distortion	Intermediate-grade DCIS	Yes
4	Blood	Discrete lesion	High-grade DCIS	Yes
5	Blood	Dilated ducts	High-grade DCIS	No
6	Blood	Nil	Invasive ductal carcinoma	No
7	Blood	Dilated ducts	Invasive ductal carcinoma	No

There were seven cases of intermediate to high-grade DCIS or carcinoma following TDE.

DCIS = ductal carcinoma in situ; TDE = total duct excision.

### Histopathology results of TDE

In total, 236 (91.1%) patients who underwent TDE were diagnosed with benign disease: 91 cases were benign papillomas, 83 were duct ectasia, 61 were benign breast changes and there was 1 myofibroblastoma. Fourteen patients (5.4%) had atypical changes including ten cases of ADH and four cases of LCIS. Nine patients (3.5%) were diagnosed with malignant disease including two cases of low-grade DCIS, three cases of intermediate-grade DCIS, two cases of high-grade DCIS and two invasive ductal carcinomas.

### Imaging

In total, 256 cases (98.8%) underwent preoperative imaging, and 65.6% of cases had an abnormality on imaging. All imaging abnormalities were associated with an increased chance of atypical changes or malignant disease on TDE, in comparison with the normal imaging group (Table 2). Any imaging abnormality, dilated ducts or the presence of a discrete lesion did not significantly increase the risk of atypical changes or malignant disease. Breast asymmetric density significantly increased the risk of atypical changes or malignant disease on TDE compared with normal imaging (*p* = 0.044). All cases of DCIS had abnormalities on imaging (*n* = 7): three were associated with dilated ducts, three had a focal lesion and one was associated with breast asymmetric density. Of the invasive carcinomas, one had normal imaging and the other had dilated ducts on ultrasound imaging.

**Table 2:** Rates of atypical changes/malignant pathology on TDE in patients with imaging abnormalities

Imaging findings	Number of cases with atypical changes/malignant disease (%)	Number with abnormal biopsy (%)	Odds ratio (95% CI)	*p*-value
No imaging abnormality	5/88 (6)	1 (1)	1	N/A
All imaging abnormalities	18/168 (11)	39 (23)	1.99 (0.71–5.6)	0.250
Discrete lesion	3/33 (9)	19 (58)	1.66 (0.37–7.4)	0.682
Dilated ducts	11/80 (14)	10 (13)	2.65 (0.88–8.0)	0.113
Breast asymmetric density or distortion	2/5 (40)	5 (100)	11.1 (1.49–82.1)	**0.044**

The table shows the number of cases of TDE that had imaging abnormalities. The odds ratio shows the chance of atypical changes/malignant disease on TDE in each imaging abnormality group in comparison with those with normal imaging. The level of significance shows the *p*-value on Fisher’s exact test when comparing the rate of atypical changes/malignant disease in each imaging abnormality group with those with normal imaging. The number of patients who had an abnormal pre-operative biopsy is also shown for each group. All abnormal biopsies were B3 lesions of abnormal or uncertain potential.

N/A = non-applicable; CI = confidence intervals; TDE = total duct excision.

Thirty-nine patients (23.2%) with an imaging abnormality had a pre-operative biopsy result of abnormal or uncertain potential on histology (B3). No B4 or B5 lesions were detected pre-operatively.

### Patients with atypical cells, preinvasive disease or malignancy

Ten patients were diagnosed with ADH and four with LCIS. This group were followed-up with annual mammography for 5 years. Two patients were diagnosed with low-grade DCIS on TDE. Three patients were diagnosed with intermediate-grade DCIS on TDE (Table 1). High-grade DCIS was diagnosed in two patients (Table 1). Invasive ductal carcinoma was identified for two patients (Table 1). Patients with a diagnosis of DCIS or carcinoma were treated with further treatment as per our breast oncology MDT recommendation.

### Risk factors for DCIS and breast carcinoma

Univariate analysis found that blood-stained nipple discharge was associated with a significant increase in the risk of intermediate- to high-grade DCIS or breast carcinoma compared with all other nipple discharge colours (*p* = 0.043), whereas clear nipple discharge was not (*p* = 0.36). The mean age of patients with DCIS or carcinoma was 67 years (range 43–81). On logistic regression, increasing age was associated with a significantly increased risk of intermediate- to high-grade DCIS or carcinoma on TDE (*p* = 0.007, *R*^2^ = 0.173).

### Procedure failure and complications

In total, 14.2% of patients who underwent TDE for nipple discharge experienced a recurrence of symptoms. This figure rose to 30.8% for those undergoing a repeat TDE procedure. The most common complications following TDE were infection (11.2%), poor wound healing (7.0%), haematoma (4.6%) and cosmetic dissatisfaction (4.2%).

## Discussion

### Histopathology results of TDE

Most patients who underwent TDE within this series were found to have benign histopathology. Papilloma and duct ectasia were the most frequent diagnoses and are already a well-established benign cause of nipple discharge.^13,14^ The incidence of breast malignancy on TDE greatly varies in the literature.^2-8^ Within this study, 3.5% of patients had a diagnosis of DCIS or carcinoma.

In a large series of 915 cases of selective duct excision, Montroni *et al* reported a much higher malignancy rate of 23%.^7^ During this study, all patients were investigated pre-operatively with nipple discharge cytology and galactography, which may have led to more careful selection of surgical candidates, increasing the rate of malignancy detection. It is expected that TDE without additional pre-operative nipple discharge studies yields a much lower malignancy rate, which is predicted to be around 3% in the ABS nipple discharge guidelines.^1^ Supporting this, a prospective study looking at 10,000 breast service referrals in New Jersey, USA, found that only 3.77% of patients who presented with nipple discharge had an underlying diagnosis of breast carcinoma.^4^ Furthermore, a UK study of 86 patients by Richards *et al*, found a malignancy rate of 2.3%. Only two patients who underwent TDE had a diagnosis of DCIS, and there were no cases of established breast carcinoma.^14^

There are several retrospective studies within the literature looking at the rate of breast malignancy following TDE. Variations in the incidence may depend on surgical criteria, nipple discharge colour, age and preoperative investigations. Risk factors for malignancy should be stratified by clinicians to decide when TDE is performed for patients with nipple discharge.

### Blood-stained nipple discharge as a marker of malignancy

ABS guidelines advise that cases of single-duct, blood-stained or clear nipple discharge should be considered for TDE owing to the risk of breast malignancy.^1^ Interestingly, all cases of intermediate- to high-grade DCIS or malignancy in this series were associated with blood-stained nipple discharge, and none with clear nipple discharge (Table 1). Foulkes *et al* found when reviewing 194 duct excision operations that all malignancies presented with blood-stained nipple discharge.^15^ Furthermore, a meta-analysis of 3,110 cases of breast cancer found that patients with blood-stained nipple discharge are at significantly greater risk of breast malignancy than those with clear discharge (*p* = 0.011) or without discharge (*p* < 0.001).^16^

Blood-stained nipple discharge may be more likely to be associated with DCIS or malignancy than other nipple discharge colours. Nipple discharge colour should always be considered in patients who may undergo TDE.

### Age as a risk factor for malignancy

It is well-established that age is an independent risk factor for breast malignancy.^17^ Here, we found that age also increases the risk of DCIS and breast carcinoma in patients with nipple discharge (*p* = 0.007). Furthermore, all patients with a diagnosis of DCIS and breast malignancy were above the age of 40.

Given that TDE can effect a patient’s future ability to breastfeed, age should be considered prior to surgical investigation of nipple discharge. It would not be unreasonable to consider a higher threshold for surgical intervention in young women with nipple discharge, in the absence of breast imaging abnormalities, and considering a microdochectomy rather than TDE.

### Imaging in nipple discharge

All imaging abnormalities increased the odds of atypical changes or malignant disease on TDE and breast asymmetric density increased the odds by 11-fold. The sensitivity of conventional ultrasound scanning and mammography breast imaging is reduced for patients presenting with nipple discharge, because causative lesions are often small and non-calcified.^18,19^ In this study, one patient with normal ultrasound scanning and mammography was later found to have invasive ductal carcinoma on TDE. One of the limitations of this study is that we have not directly compared the site of imaging abnormalities (for example the site of a lesion) with pathological outcome following TDE. Further work is required within this field to assess if the location of a breast lesion is correlated with the risk of breast malignancy in a patient group with persistent nipple discharge.

Although not routinely used, there is increasing evidence that breast magnetic resonance imaging (MRI) may be a useful adjuvant in the investigation of suspicious nipple discharge. MRI may detect DCIS in some cases that are undetectable on mammogram. In one UK-based study, bilateral breast MRI was carried out in 82 patients with persistent nipple discharge prior to diagnostic microdochectomy. Microdochectomy led to the detection of malignancy in 14 patients who had a normal mammogram and ultrasound scan. The sensitivity and specificity of MRI was 85.7% and 98.5%, respectively.^20^

### Complications of TDE and recurrence of symptoms

The most frequent complications after TDE were infection and pain, which are reported elsewhere within the literature;^21^ however, very few studies objectively quantify complication risk following TDE. A retrospective study of 915 patients who received selective duct excision for single-duct nipple discharge, reported exceptionally low complication rates of infection (*n* = 2), haematoma (*n* = 2) and nipple necrosis (*n* = 0).^7^ It is important to note that this study was performed retrospectively from a ‘breast database’, and the completeness of this analysis is unknown. In our study, all clinic letters, microbiology results and emergency department admission documentation were reviewed for each patient, to ensure that most complications were recorded. Furthermore, this study only reviews patients who underwent selective duct excision for single-duct nipple discharge; whereas we have reviewed patients who underwent TDE for any form of persistent nipple discharge. Clearly more research is needed to further quantify complication risks after nipple surgery. Further studies comparing complication rates following selective and total duct excision would also be beneficial.

Smoking history was not routinely recorded as part of this study; however, smoking is a known risk factor for breast sepsis and periductal mastitis,^22,23^ hence higher complication rates are expected within this group.

High rates of discharge recurrence were seen within this study (14%), especially for those undergoing repeat TDE (31%). In comparison, a retrospective study of 152 patients who underwent surgical treatment of periductal mastitis, found that 7.2% of patients developed recurrent mastitis at a median follow-up time of 3 years. This centre used a range of surgical techniques including a wide surgical excision, fistulectomy and extended excision with transfer of a random breast dermo-glandular flap.^8^

## Conclusions

Benign breast pathology is the most common cause for persistent nipple discharge, and papilloma is the commonest histological finding on TDE followed by duct ectasia. In this study, DCIS or malignancy was diagnosed in 3.5% of cases of TDE, and blood-stained nipple discharge was found to be significantly associated with DCIS or malignancy on TDE (*p* = 0.043). Despite this, the majority of blood-stained nipple discharge was due to benign disease, no patients with clear discharge were found to have DCIS or malignancy, and no cases of DCIS or malignancy were diagnosed on TDE in patients under 40 years of age within this series. Nipple discharge reoccurs in 14% of patients undergoing TDE and 31% of those undergoing repeat TDE.

## Data availability

The data sets generated are not publicly available to protect patient confidentiality.

## References

[1] Association of Breast Surgery. *Guidelines for the investigation and management of spontaneous nipple discharge in the absence of a breast lump*; 2019. https://associationofbreastsurgery.org.uk/media/64951/abs-summary-statement-nipple-discharge-v1.pdf (cited June 2024).

[2] Sajadi-Ernazarova KR, Sugumar K, Adigun R. *Breast Nipple Discharge*. Treasure Island: StatPearls Publishing; 2019. 28613688

[3] Parthasarathy V, Rathnam U. Nipple discharge: An early warning sign of breast cancer. *Int J Prev Med* 2012; **3**: 810–814. 23189234 PMC3506094

[4] Seltzer MH. Breast complaints, biopsies, and cancer correlated with age in 10,000 consecutive new surgical referrals. *Breast J* 2004; **10**: 111–117. 15009037 10.1111/j.1075-122x.2004.21284.x

[5] Fajdić J, Gotovac N, Glavić Z *et al.* Microdochectomy in the management of pathologic nipple discharge. *Arch Gynecol Obstet* 2011; **283**: 851–854. 20458490 10.1007/s00404-010-1481-6

[6] Dillon MF, Mohd Nazri SR, Nasir S *et al.* The role of major duct excision and microdochectomy in the detection of breast carcinoma. *BMC Cancer* 2006; **23**: 164. 10.1186/1471-2407-6-164 PMC1539014 16796740

[7] Montroni I, Santini D, Zucchini G *et al.* Nipple discharge: is its significance as a risk factor for breast cancer fully understood? observational study including 915 consecutive patients who underwent selective duct excision. *Breast Cancer Res Treat* 2010; **123**: 895–900. 20354781 10.1007/s10549-010-0815-1

[8] Zhang Y, Zhou Y, Mao F *et al.* Clinical characteristics, classification and surgical treatment of periductal mastitis. *J Thorac Dis* 2018; **10**: 2420–2427. 29850148 10.21037/jtd.2018.04.22 PMC5949503

[9] Taffurelli M, Pellegrini A, Santini D *et al.* Recurrent periductal mastitis: surgical treatment. *Surgery* 2016; **160**: 1689–1692. 27616631 10.1016/j.surg.2016.06.048

[10] Moriarty AT, Schwartz MR, Laucirica R *et al.* Cytology of spontaneous nipple discharge–is it worth it? performance of nipple discharge preparations in the college of American pathologists interlaboratory comparison program in nongynecologic cytopathology. *Arch Pathol Lab Med* 2013; **137**: 1039–1042. 23899058 10.5858/arpa.2012-0231-CP

[11] Simpson JS, Connolly EM, Leong WL *et al.* Mammary ductoscopy in the evaluation and treatment of pathologic nipple discharge: a Canadian experience. *Canadian Journal of Surgery* 2009; **52**: E245. PMC2792391 20011159

[12] Hadfield J. Excision of the major duct system for benign disease of the breast. *Br J Surg* 1960; **47**: 472–477. 13830762 10.1002/bjs.18004720504

[13] King TA, Carter KM, Bolton JS, Fuhrman GM. A simple approach to nipple discharge. *Am Surg* 2000; **66**: 960–965. 11261625

[14] Richards T, Hunt A, Courtney S, Umeh H. Nipple discharge: A sign of breast cancer? *Ann R Coll Surg Engl* 2007; **89**: 124–126. 17346403 10.1308/003588407X155491 PMC1964556

[15] Foulkes RE, Heard G, Boyce T *et al.* Duct excision is still necessary to rule out breast cancer in patients presenting with spontaneous bloodstained nipple discharge. *Int J Breast Cancer* 2011; 2011: 495315. 10.4061/2011/495315 PMC3262583 22295227

[16] Chen L, Zhou WB, Zhao Y *et al.* Bloody nipple discharge is a predictor of breast cancer risk: a meta-analysis. *Breast Cancer Res Treat* 2012; **132**: 9–14. 21947751 10.1007/s10549-011-1787-5

[17] Sun YS, Zhao Z, Yang ZN *et al.* Risk factors and preventions of breast cancer. *Int J Biol Sci* 2017; **13**: 1387–1397. 29209143 10.7150/ijbs.21635 PMC5715522

[18] Bahl M, Baker JA, Greenup RA *et al.* Diagnostic value of ultrasound in female patients with nipple discharge. AJR A. As a non-invasive and quick imaging modality, it should be considered as a useful diagnostic tool in all patients with nipple discharge. *AJR Am J Roentgenol* 2015; **205**: 203–208. 26102400 10.2214/AJR.14.13354

[19] Paula IB. Campos AM breast imaging in patients with nipple discharge. *Radiol Bras* 2017; **50**: 383–388. 29307929 10.1590/0100-3984.2016.0103 PMC5746883

[20] Zacharioudakis K, Kontoulis T, Vella JX *et al.* Can we see what is invisible? The role of MRI in the evaluation and management of patients with pathological nipple discharge. *Breast Cancer Res Treat* 2019 Nov; **178**: 115–120. 31352554 10.1007/s10549-019-05321-w PMC6790184

[21] Hagag MG, Elmeligy MH, Elkased AF. A modified technique for a common problem after major duct excision. *EJS* 2018; **37**: 330–334.

[22] Silverstein P. Smoking and wound healing. *Am J Med* 1992; **93**: S22–S24. 10.1016/0002-9343(92)90623-j 1323208

[23] Bundred NJ, Dover MS, Aluwihare N *et al.* Smoking and periductal mastitis. *BMJ* 1993; **307**: 772–773. 8219949 10.1136/bmj.307.6907.772 PMC1696425

